# Nanotechnology for Pediatric Retinoblastoma Therapy

**DOI:** 10.3390/ph15091087

**Published:** 2022-08-31

**Authors:** Eleonora Russo, Andrea Spallarossa, Bruno Tasso, Carla Villa, Chiara Brullo

**Affiliations:** Section of Medicinal and Cosmetic Chemistry, Department of Pharmacy, University of Genova, Viale Benedetto XV, 3-16132 Genova, Italy

**Keywords:** retinoblastoma, conventional chemotherapy, cancer, pediatric rare disease, nanoparticle, nanomedicine

## Abstract

Retinoblastoma is a rare, sometimes hereditary, pediatric cancer. In high-income countries this disease has a survival rate approaching 100%, while in low- and middle-income countries the prognosis is fatal for about 80% of cases. Depending on the stage of the disease, different therapeutic protocols are applied. In more advanced forms of the disease, surgical removal of the entire globe and its intraocular contents (enucleation) is, unfortunately, necessary, whereas in other cases, conventional chemotherapy is normally used. To overcome the side-effects and reduced efficacy of traditional chemotherapic drugs, nanodelivery systems that ensure a sustained drug release and manage to reach the target site have more recently been developed. This review takes into account the current use and advances of nanomedicine in the treatment of retinoblastoma and discusses nanoparticulate formulations that contain conventional drugs and natural products. In addition, future developments in retinoblastoma treatment are discussed.

## 1. Retinoblastoma

Retinoblastoma (RB) is a rare pediatric cancer of the retina. In 40% of cases, this disease is hereditary and mostly characterized by a bilateral form, whereas 60% of affected individuals have non-hereditary RB, also influenced by genetic components, with a slower course in the unilateral form. The hereditary disease occurs by a genetic mutation of the RB1 gene located at 13q14.2 chromosome [1] with the initial form of retinoma, a non-malignant tumor in the retina, visible only through optical coherence tomography. Only 5% of children show retinoma without retinoblastoma. The tumor grows toward the vitreous and/or subretinal space forming a white mass, which spreads into the vitreous in the form of seeds. The primary tumor is vascularized, while the seeds are avascular [2]. RB diagnosis is linked to some signs such as leukocoria (white pupil), reflection in the child’s eye and strabismus (misaligned eyes). In advanced disease, changes in the color of the iris and enlarged eyes, due to increased eye pressure, normally occur [3]. The diagnosis of RB cannot be carried out by direct biopsy to avoid the spread of the disease outside the eye and the formation of metastasis [4]. Indirect ophthalmoscopy with the pupil pharmacologically dilated is usually sufficient to identify the disease. Techniques such as ocular ultrasonography (β-scan) can be used to detect calcifications characteristic of RB, whereas MRI is necessary to evaluate optic nerve invasion and the presence of trilateral retinoblastoma. Unfortunately, computed tomography is not recommended, because radiation induces secondary tumors in patients with RB1 mutations [5,6].

**Figure 1:**
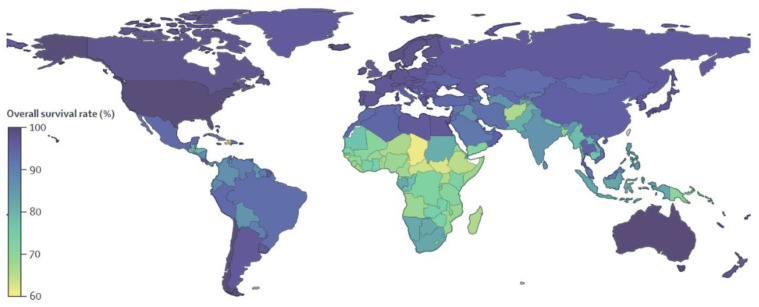
Global Prediction of Retinoblastoma Survival [10].

As evinced in Figure 1, in high-income countries this disease has a survival rate approaching 100% [7], while in low- and middle-income countries the prognosis is fatal for about 60% of cases [8,9,10].

RB classification is necessary to properly manage therapy and predict outcome. The first classification was introduced by Reese-Ellsworth in 1963 [11], followed by Murphree’s international (IIRC) classification in 2005 (Table 1) [12] which categorizes this eye tumor into five groups (from A to E, where E is the most severe Table 1) [13]; finally, Children’s Oncology Group (COG) made some minor changes to this classification [14].

Depending on the stage of the disease, different therapeutic protocols can be adopted. Local treatments (e.g., laser, photocoagulation, cryotherapy, transpupillary thermotherapy and brachytherapy) are often associated with systemic chemotherapy. In more advanced forms of the disease, surgical removal of the entire globe and its intraocular contents (enucleation) is unfortunately necessary [15].

Conservative treatment or primary enucleation are indicated according to the patient’s clinical status. In particular, specific cures can be applied to all patients with group A, B, C and D mono or bilateral retinoblastoma, whereas patients with group E bilateral retinoblastoma may be given conservative treatment. The conservative treatment relies on conventional chemotherapy (i.e., Melphalan, Topotecan, Carboplatin, Vincristine and Etoposide, see Table 2) at various dosages and durations according to the intraocular stage; this plan is generally associated with intensive and early focal treatments (e.g., brachytherapy) [16]. However, conventional anticancer drugs have many side effects associated with their use [17], and their efficacy is reduced by their being unable to overcome the blood-retinal barrier (BRB), a physiologic barrier that regulates ion, protein, and water flux into and out of the retina [18]. To overcome this problem, intra-arterial chemotherapy has normally been applied [19,20,21,22]. This technique consists of passing a micro-catheter through the femoral artery up to the ophthalmic artery of the eye and infusing chemotherapy drugs in a pulsatile way [23]. This type of administration leads to several complications, such as vitreous hemorrhage, branch retinal artery obstruction, ophthalmic artery spasm with reperfusion, ophthalmic artery obstruction, partial choroidal ischaemia, optic neuropathy and, in some cases, the death of the patient [24,25]. Because of these complications, ocular cancer therapy is normally administered through systemic, topical, sub-conjunctive and intra-vitreal delivery. In particular, nano-delivery systems that ensure a sustained drug release and that manage to reach the target site have recently been developed; in this way, the frequency of administrations can be decreased, with consequent reduction of the side effects.

This review takes into account the current use and advances of nanomedicine in the treatment of retinoblastoma and discusses nanoparticulate formulations that contain conventional drugs and natural products. In addition, future developments in retinoblastoma treatment will be discussed.

## 2. Retinoblastoma Conventional Treatment

Developed in 1990, RB therapy was based on the association of beam radiotherapy (EBRT) and systemic chemotherapy, usually combined with aggressive focal therapies [26]. In particular, EBRT was widely used until the early 2000s [27], but recently its use has been limited due to the onset of a number of side-effects, such as subsequent primary neoplasms, leukemia [28,29], and ototoxicity [30]. For these reasons, in the last ten years selective ocular delivery systems able to increase drug concentration at the ocular level and thereby reduce side-effects have been developed. In particular, ophthalmic artery chemosurgery (OAC) and eye-directed therapy, such as intravitreous chemotherapy (IVi), have deeply changed RB treatment [31,32], improving drug efficacy and reducing side-effects for patients. With the use of these innovative techniques, major improvement in eye preservation has been also obtained [33]. Currently, RB therapy is tuned according to the tumor localization (i.e., intraocular and/or extraocular disease) and it aims to preserve vision. For patients with intraocular disease, particularly those with bilateral eye involvement, a conservative approach, consisting of tumor reduction with OAC or IVi, coupled with aggressive local therapy, may result in high ocular salvage rates. To date, radiation therapy is usually reserved for cases of intraocular or extraocular disease progression [34].

### 2.1. Drug Discovery for Retinoblastoma Treatment

Despite the advances in RB therapy, in some refractory cases, this pathology remains difficult to treat. Thus, the discovery of novel drugs able to treat intraocular tumors, as well as disseminated retinoblastoma, is currently pursued by the scientific community through two major approaches: (i) repositioning of compounds already approved for other cancers [35]; and (ii) cell-based high-throughput screening (HTS) of large compound libraries. Pharmacokinetic properties (such as solubility, metabolism, ability to cross the blood-ocular or blood–brain barrier) are considered fundamental for drug repositioning, as well as for the development of drug-delivery systems. HTS has been applied, preferably testing compounds on primary cell lines derived from intraocular and cerebrospinal fluid (CSF) metastatic retinoblastoma. To evaluate drug efficacy in humans, animal models bearing tumors xenografted from intraocular or metastatic patient retinoblastomas could provide an efficient tool [36].

The identification of new intracellular targets de-regulated in retinoblastoma cancer cell lines can potentially lead to the identification of novel ani-RB agents. In this regard, Bcl-2 proteins, bromodomain and extra-terminal motif proteins (BET), MDM2/MDM4 inhibitors NF-kB, histone deacetylase, kinesin spindle protein, STAT3, cyclin dependent kinase (CDK) 4 and 6, *p*-53, MYC and GABA receptors pathways proved to be altered in RB [37,38]. The identification of compounds able to selectively block only hyperactivated or de-regulated signaling cascades in specific cancer cells could lead to targeted therapy and could represent, together with immunotherapy, a fruitful therapeutic option [33].

### 2.2. Drugs in Clinical Trials

Some examples of repositioned drugs in clinical development or in study are reported in Table 2. In most cases, these anticancer drugs are widely used in a number of solid tumors and act with different mechanisms of action. In fact, carboplatin and cisplatin are metallating agents, melphalan is an alkylating substance, doxorubicin has an intercalating action, vincristine acts on the microtubule system, blocking cell replication, whereas etoposide, topotecan and the aforementioned doxorubicin block topoisomerase, affecting cell proliferation. All of these drugs, although very effective, have serious side effects associated with their use, the impact of which can be limited by the efficient drug delivery systems. On the contrary, Palbociclib is a more recent and innovative drug, able to block an intracellular pathway involved in the inactivation of the retinoblastoma protein (pRb), a tumor suppressor that restrains G1- to S-phase progression.

To date 124 clinical trials on retinoblastoma treatment are ongoing, and 54 are completed or terminated [39]. As reported in Table 2, the most studied compounds are carboplatinum (34 studies), vincristine (22 studies), etoposide (26 studies), topotecan (17 studies) and melphalan (18 trials), often studied together or in comparison.

In trial NCT00980551, the combination of carboplatin with vincristine and topotecan is studied against recurrent or refractory RB. The endpoints of this study are: (1) to decide if the drug combination is a useful treatment for recurrent or refractory retinoblastoma; (2) to test the safety of the drug combination and to see what kind of effects (good and bad) can be expected from the drug combination; (3) to measure visual changes before and after the study therapy; (4) to use a special MRI scan to measure brain function involved in vision processing, both before and after the study therapy.

Trial NCT00110110 (phase 2, active, not recruiting) will evaluate the ability of cyclosporine to reduce drug resistance to antitumor drugs (e.g., carboplatin, etoposide, and vincristine) and allow the RB tumor cells to be killed.

Palbociclib (Ibrance, Pfizer) is one of the compounds most recently studied for retinoblastoma treatment. It is a heteroaromatic structure (6-acetyl-8-cyclopentyl-5-methyl-2-((5-(piperazin-1-yl)pyridin-2-yl)amino)pyrido [2,3-d]pyrimidin-7(8H)-one, Table 2), approved in 2017 for the treatment of HR-positive and HER2-negative breast cancer. In detail: Palbociclib selectively inhibits the cyclin-dependent kinases CDK4 and CDK6. In this manner, Palbociclib stops cell cycle progression and suppresses tumor growth [40]. Different studies have demonstrated that CD4/6 are able to phosphorylate and inactivate the retinoblastoma protein (pRb), thereby confirming the importance of CD4/6 inhibitors for different solid cancers [41,42]. In addition, its high selectivity has been shown to enhance the efficacy of other anticancer therapies and delay resistance onset [43]. Specifically, Palbociclib is currently being evaluated in two trials; one of them (phase 2, NCT01291017) involving 19 patients, is now complete and had as its purpose the determination of the efficacy and the toxicities of drug in patients with Stage IV non-small cell lung cancer with wild-type pRb. The other trial (NCT01320592) is a completed single arm, open-label, phase 1 study, in which the drug is administered to patients with pRb-expressing metastatic breast cancer in association with paclitaxel.

### 2.3. Natural Products

**Figure 2:**
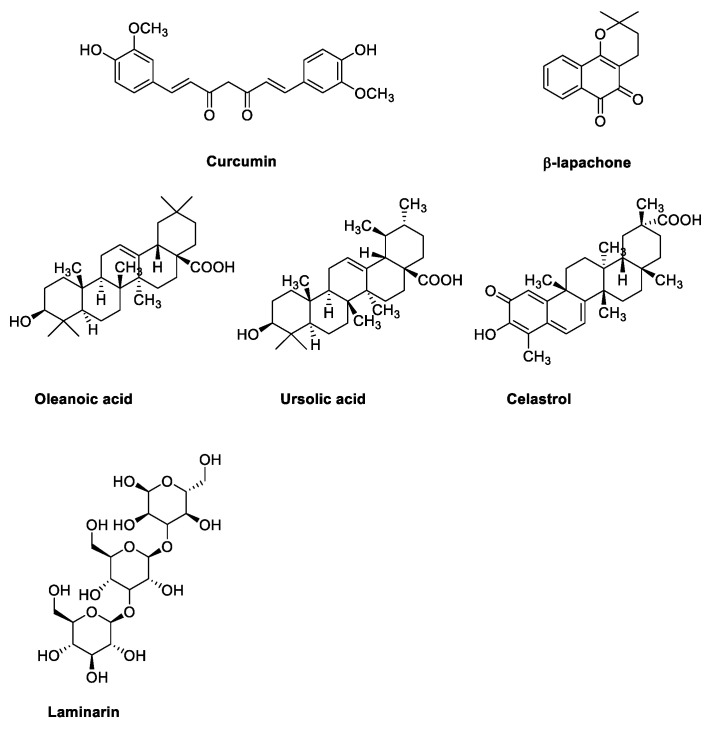
Chemical structure of natural products selected for use in retinoblastoma nanoparticles.

In addition to traditional anticancer drugs, in the last several years, a number of natural products have been investigated to treat RB. Particularly, sterol derivatives (such as oleanoic and ursolic acid), cathecol derivatives (such as curcumin) and naphthoquinone (as β-lapachone) have been studied for their interesting pharmacological activities (Figure 2). For example, β-lapachone (also named ARQ-501) is an ortho naphthoquinone originally isolated from a tree whose extract has been used medicinally for centuries. Recent investigations suggest its potential applications against numerous diseases, and particularly against cancer; its mechanism of action is not entirely known, although its ability to inhibit topisomerase enzymes has been confirmed in several studies [44]. To date, β-lapachone is under investigation in fourteen clinical trials, especially for solid tumors (e.g., head and neck neoplasms, pancreatic cancer, adenocarcinoma), but also for lymphoma [39].

Curcumin is a bright yellow chemical produced by plants of the *Curcuma longa* species. Chemically, curcumin is characterized by an α,β-unsaturated β-diketone system substituted with two phenolic functionalities. Because of its hydrophobic nature, curcumin is poorly soluble in water, and for this reason, its use as a drug is difficult. On the other hand, curcumin is normally used as an ingredient in dietary supplements, and also for cosmetic application. In the last several years its biological properties have been deeply investigated and, in particular, its anticancer activity has aroused the interest of several researchers [45,46]. To date, curcumin has been investigated in 289 clinical trials, out of which 74 evaluate the potentialities of this natural compound for the treatment of cancer [39].

## 3. Nanoparticles for Treatment of Retinoblastoma

Ocular cancer therapy has exploited various routes of administration, including systemic, topical, sub-conjunctival and intravitreal. These delivery routes are effective in the anterior area of the eye but have not been successful in diseases (including RB) in the posterior area of the eye [47,48].

The advent of nanotechnology in the field of drug delivery systems (DDSs) has introduced a new valid approach for the treatment of this disease. The goal of DDS is to transport, as a carrier, the encapsulated or bound active principle in order to reach the desired site for carrying out the therapeutic activity. The DDS’ characteristics include: (i) controlled release of the drug in order to keep the concentrations that are useful for the therapeutic action constant and prolonged in time, (ii) distribution in body regions that are difficult to access with the drug, (iii) enhancement of pharmacokinetic, pharmacodynamic, toxicity and immunogenicity properties of the active principle [49,50,51].

Nanoparticles (NPs), particles ranging from 1 to 100 nm, are composed of different materials and are widely used in nanomedicine due to their favorable characteristics. In fact, NPs’ small size enable penetration inside the cell, high surface area-to-volume ratio with consequent enhancement of all surface phenomena and low toxic damage to cell membranes and surrounding tissues [52].

NPs are indicated for the treatment of eye diseases, as they are able to pass the barriers of various tissues including the cornea, conjunctiva, sclera and blood-retinal barrier [49]. In the last 10 years, different type of NPs have been developed as DDSs for the treatment of RB, as detailed in the following paragraphs.

### 3.1. Inorganic Nanoparticles

NPs prepared using inorganic materials are widely preferred over organic NPs due to their adjustable size and shape, crystallinity, high surface area, ease of functionalization and high-density surface ligands attachment [53]. Inorganic particles possess peculiar optical, magnetic, catalytic, thermodynamic and electrochemical properties with additional bioactivities. Inorganic particles are classified as metallic and non-metallic NPs [54]. In the treatment of retinoblastoma disease, gold, silver, iron oxide, mesoporous silica and cerium oxide nanoparticles are the most used for anticancer drugs delivery.

#### 3.1.1. Gold Nanoparticles

One of the most recent studies involving the use of gold nanoparticles (Au-NPs) combines the synergistic effects of nanoformulation and ultrasonic hyperthermia [55]. In fact, the irradiation of AuNP with ultrasonic waves causes an increase of temperature (42 to 46 °C), which can induce a healing effect, either alone or in combination with other therapies. In this paper, Au-NPs used with ultrasonic waves increased the acoustic attenuation and caused its temperature to rise. Therefore, the injection of gold nanoparticles in the tumor with reduced ultrasound intensity and sonication time caused the same effects. Au-NPs have an average hydrodynamic diameter of about 89 nm and a Z potential of +38.6 mV measured by dynamic light scattering (DLS); the SEM images showed a spherical and smooth surface. The cytotoxicity of Au-NPs against retinoblastoma Y79 cells was preliminarily evaluated by MTT assay. The ultrasound hyperthermia of cells with and without Au-NPs was investigated and the results demonstrated that Au-NPs (at non-cytotoxic concentration) more deeply affect the cell viability (%) of retinoblastoma Y79 cells than does treatment with ultrasound hyperthermia alone.

In a second paper, Wang et al. [56] proposed the use of mesoporous gold nanocages (AuNCs) conjugated with Fe_3_O_4_ nanoparticles encapsulating muramyl dipeptide (MDP) as immunomodulator and perfluoropentane (PFP) for retinoblastoma diagnostic imaging and targeted therapy. AuNCs-Fe_3_O_4_/MDP/PFP system was characterized by transmission electron microscopy (TEM); the average diameter was approximately 159.3 nm, and the average zeta potential was −15.8 mV. The conjugation of Fe_3_O_4_ nanoparticles with AuNCs was highlighted in Fourier-transform infrared spectroscopy (FTIR) studies, and the release of MDP and PFP was carried out with dialysis bags with and without low-intensity focused ultrasound irradiation. The cytotoxicity of AuNCs-Fe_3_O_4_/MDP/PFP was confirmed in retinal pigment epithelial ARPE-19 cells and retinoblastoma Y79 cells, suggesting that the system was biologically safe and sufficiently promising for further utilization. In addition, in vivo studies (in mice for 8 weeks) were conducted to test both diagnostic and therapeutic activity of the developed NPs. The results showed that this nanoplatform facilitated cancer theranostics and was shown to have high biocompatibility and biosafety in vitro and in vivo, which accelerates its translation to the clinic.

Au-NPs are also considered an excellent means of destroying cancer cells when combined with laser therapy [57]. Darviot et al. incubated neuroblastoma Y79 cells with Au-NPs in Petri dishes containing a viscous medium constituted by hyaluronan powder and RPMI-1640 culture medium. Subsequently, each Petri dish was irradiated with an average of four pulses per cell with a nanosecond laser. After irradiation, cell death was estimated by propidium iodide or trypan blue. The study demonstrated that the proliferation of Y79 cells was significantly reduced 18 h after treatment. Moreover, the viscosity of the medium in which retinoblastoma cells were floating proved to affect the interaction between the laser and the Au-NPs, and a cellular death rate of 80% was achieved by irradiating at a fluence of 20 J cm^−2^ cell clusters with approximately 40 to 50 NPs/cell.

A genetic approach to fight RB was presented by Kalmodia et al. [58] who synthesized Au-NPs by Vitis vinefera L. to deliver human double minute 2 (HDM2) peptide and establish the therapeutic potential of Au-NP-HDM2 in retinoblastoma through functional knockdown of the HDM2 protein. This study established the functional effect of Au-NP-HDM2 on RB, using a Y79 cell line model and investigated the possible molecular mechanistic network modulating the therapeutic response. Au-NP and Au-NP-HDM2 were characterized by DLS, FTIR, X-ray photoelectron spectroscopy (XPS), and transmission electron microscopy (TEM) analysis. Zeta potential showed a decrease in negative potential with −11.4 mV in Au-NP-HDM2 and −18.2 mV in the Au-NP, which might enhance internalization of the peptide. The results confirmed that the nano-bio-conjugate is effective in suppressing retinoblastoma primarily by p53-mediated apoptosis, interfering with the ubiquitin-mediated proteolysis of p53, thereby upregulating the p53 protein.

Finally, Mitra et al. [59] have considered nanoconjugates for the delivery of nucleic acids such as small interfering RNA (siRNA) to knock-down the epithelial cell adhesion molecule (EpCAM) gene in retinoblastoma. In this research, siRNA was encapsulated in AuNP-polyethyleneimine (PEI) nanoparticles to specifically deliver siRNA to EpCAM, since this glycoprotein is highly expressed in various epithelial cancers. The size of the AuNP-PEI-EpCAM was about 115 nm, and the zeta potential was about +2 mV. The MTT assay, used for cytotoxicity on the Y79 cells, showed that there is no cellular cytotoxicity associated with treatment by AuNP or AuNP-PEI or AuNP-PEI-EpCAM. As has emerged by fluorescence microscopy and flow cytometry analyses, gold nanoparticle conjugates have entered in Y79 cells and AuNP-PEI-siRNA has been localized in the cytoplasm of the cells. This phenomenon would facilitate the effective release of siRNA in the cytoplasm, leading to an effective gene silencing. With this methodology the treatment potentially spares normal cells, while inducing specific gene silencing in cancer cells.

#### 3.1.2. Silver Nanoparticles

In the literature, the application of silver nanoparticles (Ag-NPs) in the treatment of RB is rather limited and the antiproliferative activity of Ag-NPs seems to be attributable to an overproduction of ROS which alter the normal physiological redox-regulated functions in the cells [60]. Two types of 20 nm AgNPs coated with hydrocarbon or polysaccharide were considered and tested for their efficacy on pRb, which is a critical protein that plays in this case an inhibition of the cell cycle. AgNPs seem to play a fundamental role at the genetic level by eliminating the hyperphosphorylation of pRb and therefore arresting the cell cycle progress. It was finally demonstrated that both AgNPs can induce cell cycle arrest, polysaccharide-coated nanoparticles being less toxic because they tend to be separately distributed and produce less ROS, whereas hydrocarbon-coated particles tend to agglomerate.

#### 3.1.3. Iron Oxide Nanoparticles

As previously mentioned, a study on gold nanoparticle conjugate containing Fe_3_O_4_ has already been reported [56].

Demirci et al. [61] proposed the combination of iron-containing magnetic nanoparticles and hyperthermia for the treatment of RB. Hyperthermia is a therapeutic tool used for cancer treatment; in fact, tumor cells are more sensitive to heat than are normal cells. The increase of temperature can be achieved by different techniques, including radio frequency, microwaves, and focused ultrasound. Iron nanoparticles, through a phenomenon called magnetic hyperthermia [62], have been used as nano-heaters able to target tumor cells without damaging normal tissue. The authors presented the evaluation of magnetic hyperthermia using dextran-coated iron nanoparticles in the Y79 retinoblastoma cell line. The results showed that after 24 h of magnetic hyperthermia treatment was induced 46% to 73% of death in Y79 retinoblastoma cells. Cell death was observed with 0.75 and 1 mg/mL of nanoparticle concentration at 24 and 72 h after magnetic hyperthermia and a maximum tumor cytotoxicity at 24 h with a concentration of 0.75 mg/mL. The study highlighted that magnetic hyperthermia with dextran-coated iron nanoparticles can be a promising therapeutic option for retinoblastoma.

#### 3.1.4. Mesoporous Silica Nanoparticles

Mesoporous silica nanoparticles (MSNs) are biodegradable inorganic nanomaterials that can degrade into silicic acid or silica species. Due to their widely accepted biocompatibility, they are considered one of the most promising platforms for biomedical applications, such as drug delivery. This type of nanoparticle was investigated for the treatment of retinoblastoma and the most significant studies are reported below.

Qu et al. [63] presented a drug delivery system consisting of Carboplatin (CRB) loaded in the MSNs, which contain the carboxylic acid (COOH) group on the outer surface. The acid functionalities can conjugate the amine group of epithelial cell adhesion molecule (EpCAM), a transmembrane protein overexpressed in the retinoblastoma. The so-obtained nanoconjugate (EpCMSN) was characterized technologically for particle size, shape and drug release abilities. The average particle size of EpCMSN was 148.5 ± 2.35 nm with a uniform dispersion index of 0.15, zeta potential was −20.1 ± 2.15 mV and the particle shape, confirmed by TEM imaging, was perfectly spherical and smooth, indicating the success of formulation technique. The in vitro release of CRB was estimated by the dialysis method, which provided a controlled release profile. About 30% of encapsulated drug was released within 24 h while 80% of drug was released after 100 h. There was no burst release, which indicates that the entire drug was encapsulated in nanoparticles. Then biological assays were performed to evaluate the targeting efficiency (cellular uptake analysis in retinoblastoma cells), cytotoxicity (MTT assay) and apoptosis (caspase-3 assay). The cellular uptake of EpCMSN was good, resulting more effective than the free drug in inhibiting cell proliferation (IC_50 (EpCMSN)_ = 1.38 μg/mL; IC_50 (CRB)_ = 3.26 μg/mL). Furthermore, EpCMSN enhanced apoptosis of cancer cells in retinoblastoma cells (2-fold increase in caspase-3 level compared to that of free CRB).

In another paper [64], MSNs were grafted with the antibodies anti-MRC2 and/or anti-CD209 for retinoblastoma photodynamic therapy (PDT). The cellular uptake of these MSNs occurred via endocytosis and the co-localization into the lysosomes was monitored by confocal imaging on living retinoblastoma cell lines. Irradiation was performed with a confocal microscope with a focused laser beam and at maximum laser power. The photodynamic efficiency indicated that MSN-CD209 and MSN-MRC2 were able to induce significant toxicity with about 20% to 25% of cell death for Y-79 retinoblastoma cells. The data evinced the pharmacological potential of targeted mesoporous silica nanoparticles for retinoblastoma treatment by a non-invasive method with reduced side effects.

Similar work was presented by Gary-Bobo et al. [65] in which multifunctionalized MSNs were obtained by combining a one-photon excitation photodynamic therapy (OPE-PDT) agent, camptothecin (CPT) as anticancer drug, mannose or galactose for targeting retinoblastoma cells Y-79. Confocal microscopy demonstrated that the endocytosis of the MSN was mediated by carbohydrate receptors. Promising results were observed with MSNs functionalized with mannose, since a strong cell death was obtained after only a short irradiation at a low fluence. Furthermore, MSNs containing camptothecin combined with OPE-PDT were identified as a promising therapeutic synergy to kill cancer cells.

Finally, Warther et al. [66] reported the study on 25 nm diameter MSNs functionalized with mannose for their use to retinoblastoma cell targeting and imaging. MSNs were prepared introducing, in optimal quantity, polyethylene glycol (PEG) to avoid aggregation since mannose alone was not able to stabilize silica nanoparticles. Once the MSNs were stabilized, zeta potential in water was measured and was slightly positive (+6 mV), in agreement with the substitution of negative Si-O groups by neutral PEG and mannose. Y-79 retinoblastoma cells were incubated at 37 °C for 5 h with MSN (concentration 40 mg/mL) and after incubation the localization of nanoparticles was analyzed by fluorescence microscopy experiments. MSN nanoparticles were almost always localized within lysosomes, thus indicating an entry in the cells through an endocytosis pathway.

#### 3.1.5. Cerium Oxide Nanoparticles

Cerium is the first element of the lanthanide and existed as cerium oxide in both CeO_2_ and Ce_2_O_3_ oxidation forms. Cerium oxide nanoparticle expose on their surface a mixture of cerium (III) and cerium (IV) and the pharmacological basis of the nanoparticle’s activity was related to its ability to absorb and release oxygen [67].

Cerium oxide nanoparticles (CNPs) have attracted great attention in the field of nanotechnology due to their antioxidant and anti-inflammatory properties [68]. As oxidative stress induced by ROS is associated with a number of human pathological disorders, CNPs can be a potential therapeutic option for the treatment of various acute and chronic diseases [69].

In recent years, two interesting papers have been published highlighting the use of CNPs for RB therapy [70,71].

Gao et al. [70] reported a new nanocarrier consisting of glycol chitosan-coated cerium nanoparticles (GCCNPs) as a pH-responsive and controlled DDS able to deliver a CXCR4 antagonist (AMD11070), and doxorubicin (DOX) for tumor targeted and tumor microenvironment (TME) responsive combination therapy. The GCCNPs were synthesized using standard bioconjugation techniques [72], including DOX and AMD11070 through a cross linker. The size of the AMD–GCCNPs–DOX (226.1 ± 18.87 nm) was significantly (*p* < 0.05) larger than the average diameter of the GCCNPs (171.8 ± 3.71 nm) but no variation in polydispersity index was observed, and zeta potential was positive for both formulations (+23.30 ± 2.12 mV). Therapeutic efficacy was shown in RB xenograft and in genetic p107s orthotopic mice models. Positively charged NPs’ surfaces at physiological conditions (pH 7.4) may improve their transport through the negatively charged vitreous, and further enhance their internalizations into the tumor cells. This study demonstrated a synergistic strategy to improve the therapeutic efficacy and reduce the side effects of DOX with significant inhibition of tumor growth as well as biocompatibility with normal retinal cells in vivo.

More recently, Kartha et al. [71] investigated cerium-doped titanium dioxide (Ce-doped TiO_2_) nanoparticles for anticancer activity against Y79 retinoblastoma cancer cells. XRD, SEM, TEM, and photodynamic anticancer activity analyses were used to characterize these nanoparticles. TiO_2_ and Ce-doped TiO_2_ nanoparticle were incubated into Y79 retinoblastoma cancer cells and were treated with UV radiation over various periods ranging from 1 to 6 h. TiO_2_ exhibited 66.4% while Ce-TiO_2_ demonstrated 69.4% of anticancer cytotoxicity, demonstrating that cerium nanoparticles are more active against tumor cells.

### 3.2. Organic Nanoparticles

The most employed organic NPs for drug delivery in retinoblastoma therapy are represented by polymer-based nanoparticles [73,74,75] (including Polylactic-co-glycolic acid (PLGA), Chitosan (CH), Poly-Caprolactone (PCL) and Polymethylmethacrylate (PMMA) NPs), lipid-based nanoparticles (LNP) and lactoferrin nanoparticles.

In particular, Mudigunda et al. [76] recently reported hybrid PLGA/PCL NPs loaded with an anticancer drug, Palbociclib (PCB), and a photothermal dye IR820 (IR). PLGA and PCL were chosen as a delivery system owing to their biocompatibility and distinct degradation rates. Hybrid NPs were characterized in size (170–200 nm) by SEM and the entrapment efficiencies and drug loading were 80 and 18.5% in PCB and 81.5 and 20.2% in IR82, respectively. Apoptosis studies of the PCB/IR NPs in Y79 retinoblastoma cells indicated that PCB had excellent bioavailability in retinoblastoma cells. The in vitro cytotoxic efficacy of free PCB, IR, and PCB/IR NPs was also evaluated in the retinoblastoma (Y79) cell line. It was observed no significant toxicity. Furthermore PCB/IR NPs were found to be biocompatible and demonstrated excellent photothermal and photoacoustic imaging properties.

Godse et al. [77] suggested the use of PLGA nanoparticles coated with chitosan and galactose to prevent fast drug release (etoposide, ETP), stabilize the colloidal particles and help in active targeting RB cells, as they are known to express sugar receptors (lectins) that exhibit a preferential affinity for galactose residues. PLGA nanoparticles were prepared with the solvent diffusion method and conjugated with previously synthetized chitosan-galactose (CG) dimer. Then, these NPs were characterized by ETP loading (about 6%) and entrapment efficiency (71.5 ± 5.5%). The particles size of GC-PLGANPs was 158 ± 0.77 nm with polydispersity of 0.23 ± 0.045, which revealed and good uniformity and distribution, zeta potential was +25 mV, an optimal value to diffuse out of the vitreous and achieve effective retinal penetration. Furthermore, TEM analysis indicated uniform and spherical shape. The release profile of the free drug showed complete release in 6 h; moreover, GC-PLGANPs showed a sustained release up to 32 h and the kinetic release fitted the first order model (Fickian diffusion). Finally, cellular uptake study revealed the enhanced cellular internalization of GC-PLGANPs cytotoxicity and apoptosis studies showed good anti-cancer effect.

Sims et al. [78] designed and evaluated unmodified and surface modified PLGA-NPs containing melphalan for intra-arterial treatment of retinoblastoma [79]. PLGA-NPs were prepared using the emulsion solvent evaporation method and melphalan was incorporated. The surface-modified PLGA-NPs were obtained with biotinylated peptides (namely, MPG and TET1) and PEG after the surface addition of avidin palmitate. Particle size of the unmodified, MPG, PEG, and TET NPs were of 145 ± 6.54, 129 ± 6.38, 107 ± 6.64, and 123 ± 6.33 nm, respectively. The melphalan loading was of 7.3 ± 0.4 µg/mg of NPs with significant differences in melphalan loading as a function of surface-modification. To avoid poor drug loading and inadequate melphalan release, a PVA solution was employed for NP hardening and saturated with melphalan (either 1 or 10 mg/mL) to prevent unwanted diffusion from the NPs. The effect of surface modification on association and internalization in Y79 retinoblastoma cells was determined via flow cytometry. All surface-modified NPs were more adequate for internalization than unmodified NPs would have been, but MPG NPs proved to be the most efficacious formulation. This research highlighted that the surface-modified NP formulations may significantly improve retinoblastoma treatment when administered in vivo.

Garcia et al. [80] developed a new ocular delivery system of Pioglitazone (PGZ-)loaded PLGA-PEG NPs using an experimental factorial design. These nanoparticles were characterized by size (160.0 ± 1.3 nm), presented a monodispersion (PI < 0.1) and high drug entrapment efficiency (92%), which is suitable for ophthalmic application. The zeta potential was −13.9 mV, indicating an adequate short-time stability. In vitro release profile, ex vivo transcorneal and transcleral permeations and in vivo assays were performed to demonstrate that PGZ-PLGA-PEG NPs were suitable for the prevention of the ocular inflammatory process. Hen’s egg chorioallantoic membrane (HET-CAM^®^) and the Draize test were carried out to confirm that these systems do not induce eye irritation and could present novel systems to more effectively act against ocular inflammatory diseases.

Among the papers that considered chitosan and its derivatives for the preparation of nanoparticles in the treatment of retinoblastoma, two were particularly significant and important. In the first, Delrish et al. [81] investigated thiolated and methylated chitosan carboxymethyl dextran nanoparticles (CMD-TCs-NPs and CMD-TMC-NPs) for intravitreal (IVT) injection into rat eyes with retinoblastoma. CMD-TCs-NPs and CMD-TMC-NPs were characterized by particle size (34 ± 3.78 nm and 42 ± 4.23 nm, respectively) and zeta potential (+11 ± 2.27 mV and +29 ± 4.31 mV, respectively). Confocal imaging was used to track the diffusion in the vitreous cavity of the injected Cy5-labelled nanoparticles; the results showed that CMD-TMC-NPs were completely trapped in the vitreous and were unable to reach the retina even after 24 hr, due to their high positive zeta potential. The study revealed that appropriate surface charges (preferably +11 ± 2.27 mV) on the surface of NPs (CMD-TCs-NPs) enhanced the diffusion after IVT injection. In a second paper, Delrish’s research group [82] focused on thiolated chitosan nanoparticles containing topotecan (TPH-TCs-NPs). These nanoparticles were produced by ionic gelation method and sodium tripolyphosphate (TPP) was used as cross-linking agent. The entrapment efficiency (EE), drug loading (DL) and yield were 85.23 ± 2%, 12.5 ± 0.03% and 61.81%, respectively. Additionally, the size of NPs was 25 ± 2 nm (PDI: 0.21 ± 0.03) and the zeta potentials measured +12 ± 2 (mV). Higher values of zeta potentials were recorded for N-trimethyl chitosan (TMC) nanoparticles, which were also tested in this study. The release of topotecan from TCs-NPs was sensitive to glutathione (GSH) concentrations, a peptide found in cancer cell, and about 70% of the loaded topotecan was released within 16 h. Because of their adequate surface zeta potential, TPH-TCs-NPs showed improved cellular uptake and cytotoxicity in comparison to free TPH. The apoptosis study, conducted by in vitro flow cytometry, and the in vivo intravitreally injection of TPH-TCs-NPs have shown that this nanocarrier can pass through biological barriers, delivering TPH to the lesion site with elevated efficacy, contributing to the effectiveness of the cancer therapy.

Regarding the use of lipid nanoparticles (LNP), examples in the literature refer to the co-delivery of siRNA with other conventional chemotherapeutic drugs used in retinoblastoma treatment. Passos Gibson et al. [83] developed lipid nanoparticles (switchable LNP) constituting of cationic switchable lipid (CSL), 1,2-distearoyl-sn-glycero-3-phosphocholine (DSPC), Cholesterol (Chol) and N-(carbonyl-methoxypolyethyleneglycol 2000)-1,2-distearoyl-sn-glycero-3-phosphoethanolamine, sodium salt (DSPE-PEG2000), prepared by microfluidics or by manual extrusion. These LNPs were able to deliver siRNA with the cell and downregulate survivin, which is overexpressed in retinoblastoma cancer cell. Furthermore, the combined effect of surviving downregulation and incubation with standard chemotherapeutics (namely, carboplatin, melphalan, topotecan and teniposide) was evaluated to identify the antineoplastic dug to benefit the most from survivin downregulation in future clinical applications. LNPs obtained by microfluidics or extrusion were fully characterized for their size (133 ± 2 nm, 146 ± 2 nm), PI (0.175 ± 0.02, 0.101 ± 0.01), zeta potential (31.5 ± 4.1 mV, 34.3 ± 1.3 mV) and entrapment efficiency (96.37 ± 1.35%, N/A). This research highlighted that teniposide and topotecan were not significantly impacted by survivin downregulation, whereas carboplatin and melphalan showed some activity. Further studies will be focused on survivin silencing prior to chemotherapy with melphalan and/or carboplatin in a rat model of retinoblastoma. Fluorescence imaging demonstrated that switchable LNPs possessed in vitro and in vivo high affinity for retinoblastoma cells (Y79) [84]. The switchable LNPs were used as a dual delivery system for miRNA and melphalan and empowered the synergy of both drugs in human primary cells as well as in a rat model of RB.

Protein nanoparticles have several advantages as drug delivery systems, such as biodegradability, being easily metabolized, and having low toxicity and immunogenicity. Moreover, they interact very easily with both hydrophilic and hydrophobic drugs and, possessing charged groups, could be chemically modified. The apo-transferrin and lactoferrin (Lf) are iron transporting proteins, highly expressed on the surface of cancerous cells to sustain the iron demand of tumor cellular metabolism. Narayana et al. [85,86] described the preparation of carboplatin-(CPT) and etoposide-(ETP) loaded lactoferrin nanoparticles (Lf-Nps) obtained by the solution–oil chemistry method. The characterization was carried out by measuring: particle size, 61.2 ± 3.94 nm for CPT-Lf-Nps and 45.15 ± 5.85 nm for ETP-Lf-Nps; encapsulation efficiency, 59.63 ± 8.02% for CPT-Lf-Nps and 38.05 ±4.75% for ETP-Lf-Nps; drug loading for CPT and ETP was 11.92 ± 1.6% and 7.61 ± 0.95%, respectively. Moreover, the intracellular uptake of Lf-Nps was explored by loading into the nanoparticles the R6G fluorescence dye. The Lf-R6G nanoparticles entered the cells and were retained for a longer time than R6G alone. Despite the fact that the collected results are encouraging, further validation studies should be conducted to evaluate the efficacy of different drugs loaded in Lf-Nps and to assess the efficacy of this system in vivo in retinoblastoma cancer stem cell xenograft models.

Finally, polymethylmethacrylate (PMMA) polymer has been used to develop organic nanoparticles against retinoblastoma. Shome et al. [87,88] described new carboplatin-loaded PMMA nanoparticles (NPC) as a model system for the treatment of advanced intra-ocular retinoblastoma. The carboplatin-loaded PMMA nanoparticles showed nearly spherical shape, with an average diameter of 110 ± 10 nm, polydispersity index lower than 0.25, a negative zeta potential, and a biphasic pattern of carboplatin release, due to a sustained release behavior. This study established that PMMA-NPC had greater intra-ocular penetration and achieved higher intravitreal concentration than did the free carboplatin, and that it could be a promising treatment for retinoblastoma presenting vitreous seeds.

### 3.3. Nanoparticles for Natural Product

In the research of novel and more efficient treatment for RB, nanocarriers for the delivery of natural products have been developed.

In the last several years, curcumin has attracted the attention of researchers for its applications in retinal disorders. To increase and improve its bioavailability, solubility and stability in an aqueous medium, nanotechnology has been used. Alsaab et al. [89] reported a novel folate receptor-targeted drug delivery system for retinoblastoma cells containing curcumin-difluorinated (CDF), and loaded in poly(styrene-co-maleic acid)-conjugated-folic acid (SMA-FA)-CDF micelles. Nanomicelles were characterized for size (193.6 ± 20 nm), polydispersity index (0.175 ± 0.05), zeta potential (−7.12 ± 4 mV), encapsulation efficiency (EE, 75.98 ± 12%) and drug loading (DL, 12.14 ± 2.23%). TEM analysis of FA-SMA-CDF surface indicated spherically-shaped nanoparticles. To determine the efficacy of the developed formulations against different types of retinoblastoma cells, the cytotoxicity of FA-SMA-CDF was evaluated (MTT assay) on normal retinal (ARPE-19) cells and on retinal cancer cell lines (Y-79 and WERI-RB1). The results showed an increase of retinoblastoma cytotoxicity cells while demonstrating lower cytotoxicity on normal cells, indicating the nanoparticles safety as to the healthy tissue.

Nanoformulation studies have been performed on the encapsulation of celastrol, a pentacyclic nortriterpen quinone extracted from Chinese herbal medicine and endowed with anti-tumor activity [90]. Guo et al. [91,92] proposed the synthesis of the copolymer poly[thioctic acid-grafted poly(ethylene glycol)/(benzyl amine)], denoted as PTEB, for nanomicelle formulation containing celastrol (C-PTEB). This novel polymeric vector was characterized by size (69 ± 3 nm), drug loading (8.51%) and by TEM, indicating its uniform spherical shape. Y79 cell uptake of C-PTEB was evaluated with confocal laser scanning microscopy (CLSM) and the results indicated a rapid internalization and subsequent fast celastrol release. Furthermore, the results of flow-cytometric analyses showed that celastrol and C-PTEB induced apoptosis of the retinoblastoma Y79 cells, demonstrating that this nanocarrier could represent a valuable system for retinoblastoma therapy.

Silva et al. [93] studied PLGA nanoparticles loaded with oleanolic (OA) and ursolic (UA) acids as a single dosage combination for the treatment of retinoblastoma. PLGA-OA/UA nanoparticles were characterized by particle size (213.55 ± 1.60 nm), polydispersity index (0.090 ± 0.038) and zeta potential (−27.12 ± 0.27 mV). This formulation presented a high cytotoxic activity against the Y-79 cell line, and the behavior was promising for retinoblastoma treatment.

N’Diaye et al. [94] developed lipidic nanoparticles (LNPs) consisting of a poly(D,L)-lactide (PDLLA) nanoparticle coated with a phospholipid (1-palmitoyl-2-oleoyl-sn-glycero-3-phosphocholine/1,2-dioleoyl-3-trimethylammonium-propane) bilayer containing beta-lapachone (β-Lap), as antitumor drug, and a photosensitizer temoporfin, Foscan (THPC) for combined chemo- and photodynamic retinoblastoma therapy. The hybrid nanoparticles showed a particle size of 170 ± 3 nm, a polydispersity index (PDI) of 0.08 ± 0.02, a negative zeta potential value of −46 ± 1 mV, an entrapment efficiency (EE) and drug loading (DL) of β-Lap of 12.82% and 1.97%, respectively. Fluorescent LNPs were incubated with Y79 cells for different times and their kinetics of internalization were assessed by confocal microscopy; after 48 h incubation LNPs were distributed all over the cytoplasm of cells. Furthermore, β-Lap in free form or encapsulated in LNPs was identified as the only cytotoxic compound against Y79 cells. The studied system emerged to be active in both chemotherapy and photodynamic therapy and could be administered in a single intravitreal injection.

Remya et al. [95,96] conducted research on laminarin, a storage polysaccharide obtained from the brown algae *Turbinaria ornata* (*T. ornata*). The study presented a rapid, simple, cost-effective and green method for the synthesis of silver nanoparticles (AgNPs) from the brown seaweed *Turbinaria ornata.* AgNPs were characterized by particle size (ranged from 22 to 32 nm), zeta potential (−28.7 mV), total phenol content by Folin-Ciocalteu method (43 ± 2.52 mg/g GAE) and ABTS scavenging activity (144.21 µg/mL); the latter assays revealed the high antioxidant and antiproliferative activity of the samples. The antiproliferative activity of the synthesized AgNPs was evaluated against retinoblastoma cell line Y79 through studies of cytotoxicity and flow cytometry to verified cell apoptosis. The results showed that AgNPs containing laminarin exhibited strong cytotoxic effects inducing apoptosis of retinoblastoma cell lines. The obtained data confirmed the potential of this novel nanocarrier for the treatment of retinoblastoma.

## 4. Future Perspective

Emerging trends in retinoblastoma care predicted multifunctional nanoparticles integrating the early diagnosis and treatment.

Zou et al. [97] proposed a new metal–organic framework nanoparticles (CM-NPs) coated with tuftsin (CMT NPs), which not only supported photoacoustic (PA)/magnetic resonance (MR) imaging but also had a photothermal/immunotherapeutic effect. These nanoparticles were unique magnetic carbonized carriers characterized by particle sizes of 303.1 ± 48.53 nm, zeta potential of –12.1 ± 2.88 mV, encapsulation efficiency and drug loading of 29 ± 4.09% and 2.64 ± 0.37%, respectively. In vitro studies performed on healthy ARPE-19 cells showed that CMT NPs were biologically safe and had the potential for further application in cancer therapy. Furthermore, CMT NPs, under an in vitro magnetic field, accumulated in Y79 cells with an increase of CMT NP concentration (and consequently of tuftsin) in the target area. The same behavior was verified using in vivo Y79 tumor-bearing nude mice on which were conducted fluorescence imaging. Finally, in vivo studies showed a good efficiency of the combination of laser irradiation and CMT NPs administration. In this condition, a reduction of the tumor volume was observed with an improvement of the therapeutic effect. Moreover, the results of western blotting and ELISA assays showed that laser +CMT NPs could effectively induce macrophage typing, providing a favorable platform for tumor immunotherapy.

Similar research work was presented by Li et al. [98] reporting a novel nanoplatform folate-receptor (FR) targeted laser-activatable liposome loaded with doxorubicin (DOX) and indocyanine green (ICG) and liquid perfluoropentane (PFP) for photoacoustic/ultrasound (PA/US) imaging-guided chemo/photothermal retinoblastoma therapy. This multifunctional liposome (FA-DOX-ICG-PFP@Lip) was constituted in the outer shell by 1,2-Distearoyl-sn-glycero-3-phosphoethanolamine-N-[folate(polyethyleneglycol)-2000] (DSPE-PEG (2000)-folate), 1,2-Dihexadecanoyl-rac-glycero-3-phoshocoline (DPPC), cholesterol and was produced using a double-emulsion method. FA-DOX-ICG-PFP@Lips were characterized by particle size (309.4 ± 16.7 nm), and zeta potential (–20.9 ± 8.6 mV). The encapsulating efficiency and drug loading of DOX and ICG were 62.04 ± 5.10% and 6.20 ± 0.51%, 91.85 ± 2.98% and 9.19 ± 0.29%, respectively. The promising cytotoxicity, cell uptake and anticancer effect demonstrated by in vitro evaluations supported in vivo experiments on this system. In particular, the synergistic anticancer efficacy of FA-DOX-ICG-PFP@Lip was evaluated in mice by injecting the multifunctional liposome and irradiating the tumor regions with a laser (808 nm, 1 W/cm^2^, 5 min). This treatment induced a change in the body weights of the mice, indicating the effectiveness of the new nanoplatform.

In Table 3 the combination studies of nanoparticles drug therapy with other effective therapies against RB are summarized.

Finally, it is necessary to mention the last frontier of RB treatment which involved the use of oncolytic viral vectors that can be included in the nanomedicine approach [33,99,100].

Oncolytic viral therapy utilizes genetically modified viruses capable of selective replication in tumor cells through two mechanisms of action: lysis and/or induction of systemic antitumor immunity.

In normal cells, the retinoblastoma tumor suppressor gene (RB1) binds to free E2F transcription factors, forming a complex that inhibits cell proliferation. When RB1 is phosphorylated by cyclin-dependent kinases, the inability to bind E2F occurs and consequently the cell cycle progresses from G1 to S [101]. Therefore, a strategic approach in the treatment of retinoblastoma could be to target RB1 dysfunction and increase E2F expression.

Pascual-Pasto et al. [102] showed that VCN-01, a clinical-grade oncolytic adenovirus genetically modified from adenovirus type 5 designed to replicate in cancer cells with an altered RB1-E2F pathway, was active, tumor selective, safe, and clinically translatable, providing a new valid therapeutic option to save vision in the pediatric patients.

At the same time, other authors [103,104,105] highlighted the importance of viral oncolytic therapy as a chemotherapy-independent treatment option for retinoblastoma.

## 5. Conclusions

This review connected the nanocarrier with the delivery requirements specific to retinoblastoma treatment. A large number of organic polymers or inorganic nanoparticles loaded with synthetic or natural drugs have been developed. All systems showed an increase in the bioavailability of the encapsulated drug, and an ability to pass the BRB and reduce the side effects of anticancer drugs. “Smart multifunctionalized nanosystems” (i.e., systems able to reach the complicated anatomical regions of the eye affected by retinoblastoma) represent the last frontier in this area.

## Figures and Tables

**Table 1:** International classification of intraocular retinoblastoma (IIRC).

Group Classification	Morphological Characteristics	Image of Ocular Damage
**Group A**	Small tumors (≤3 mm) confined to retina; >3 mm in fovea; 1.5 mm in optic disc	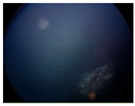
**Group B**	Tumors (>3 mm) confined to retina, clear subretinal fluid (≤6 mm from tumor margin)	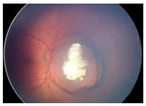
**Group C**	Localized vitreous and/or subretinal seeding (<6 mm from tumor margin)	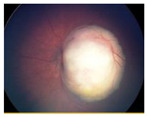
**Group D**	Diffuse vitreous and/or subretinal seeding (>6 mm from tumor margin). Subretinal fluid > 6 mm from tumor margin.	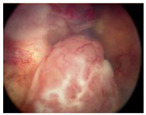
**Group E**	No visual potential and poor prognostic features; Retinoblastoma occupying > 50% of the globe, invasion of the optic nerve, choroid, sclera, orbit, anterior chamber	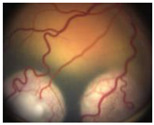

**Table 2:** Clinical trials on retinoblastoma.

Drug	Molecular Formula	N. of Trials	N. of Clinical Trials Completed or Terminated	Details
**Carboplatinum**	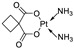	34	14	Results available for 6 studies
**Etoposide**	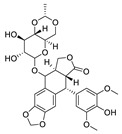	26	10	Results available for 5 studies
**Vincristine**	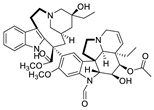	22	7	Results available for 6 studies
**Melphalan**	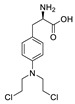	18	9	Results available for 2 studies
**Topotecan**	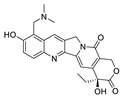	17	6	Results available for 2 studies
**Doxorubicin**	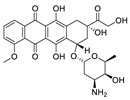	3	1	NCT00186888, active, not recruiting, phase 3; NCT01783535, recruiting, phase 2; NCT00004006, completed, phase 2.
**Palbociclib**	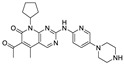	2	2	NCT01291017, phase 2; NCT01320592, phase 1.
**Cisplatinum**	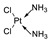	4	1	NCT00554788, phase 3; NCT03567642, phase 1; NCT00002675, phase 2; NCT00003273, phase 2

**Table 3:** Combination therapy in RB.

Drug/NPs	Combination Therapy	Ref.
Gold NPs	Ultrasonic Hyperthermia	[55]
Gold NPs	Laser therapy	[57]
Iron oxide NPs	Magnetic Hypertermia	[61]
Mesoporous silica NPs	Photodynamic therapy (PDT)	[64]
Camptothecin/Mesoporous silica NPs	Chemo/one-photon excitation photodynamic therapy (OPE-PDT)	[65]
Doxorubicin/Cerium oxide NPs	tumor targeted/tumor microenvironment (TME) therapy	[70]
Palbociclib/polymeric NPs	Chemo/photodermal therapy (PTT)	[76]
Beta-lapachone/lipidic NPs	Chemo/photodynamic therapy	[94]
Carbon-Metal-tuftsin NPs	Photothermal/Immuno therapy	[97]
Doxorubicin/Liposomes	Chemo/Photothermal therapy	[98]

## Data Availability

Not applicable.

## References

[1] Friend S.H., Bernards R., Rogelj S., Weinberg R.A., Rapaport J.M., Albert D.M., Dryja T.P. (1986). A human DNA segment with properties of the gene that predisposes to retinoblastoma and osteosarcoma. Nature.

[2] Grossniklaus H.E. (2014). Retinoblastoma. Fifty Years of Progress. The LXXI Edward Jackson Memorial Lecture. Am. J. Ophtalmol..

[3] Dimaras H., Kimani K., Dimba E.A.O., Gronsdahl P., White A., Chan H.S.L., Gallie B.L. (2012). Retinoblastoma. Lancet.

[4] Karcioglu Z.A. (2002). Fine needle aspiration biopsy (FNAB) for retinoblastoma. Retina.

[5] de Jong M.C., de Graaf P., Noij D.P., Göricke S., Maeder P., Galluzzi P., Brisse H.J., Moll A.C., Castelijns J.A. (2014). Diagnostic performance of magnetic resonance imaging and computed tomography for advanced retinoblastoma: A systematic review and meta-analysis. Ophthalmology.

[6] de Jong M.C., Kors W.A., Graaf P., Castelijns J.A., Kivelä T., Moll A.C. (2014). Trilateral retinoblastoma: A systematic review and meta-analysis. Lancet Oncol..

[7] Global Retinoblastoma Study Group (2020). Global retinoblastoma presentation and analysis by national income level. JAMA Oncol..

[8] Chantada G., Fandiño A., Manzitti J., Urrutia L., Schvartzman E. (1999). Late diagnosis of retinoblastoma in a developing country. Arch. Dis. Child..

[9] Chawla B., Hasan F., Azad R., Seth R., Upadhyay A.D., Pathy S., Pandey R.M. (2016). Clinical presentation and survival of retinoblastoma in Indian children. Br. J. Ophthalmol..

[10] Wong E.S., Choy R.W., Zhang Y., Chu W.K., Chen L.J., Pang C.P., Yam J.C. (2022). Global retinoblastoma survival and globe preservation: A systematic review and meta-analysis of associations with socioeconomic and health-care factors. Lancet Glob. Health.

[11] Reese A.B., Ellsworth R.M. (1963). The evaluation and current concept of retinoblastoma therapy. Am. Acad. Ophthalmol. Otolaryngol..

[12] Murphree A.L. (2005). Intraocular retinoblastoma: The case for a new group classification. Ophthalmol. Clin. N. Am..

[13] Rusakevich A.M., Schefler A.C. (2022). Retinoblastoma: Recent Trends in Diagnosis and Management. Curr. Surg. Rep..

[14] https://www.childrensoncologygroup.org/index.php/newlydiagnosedwithretinoblastoma.

[15] Leclerc R., Olin J. (2020). An Overview of Retinoblastoma and Enucleation in Pediatric Patients. AORN J..

[16] https://archivio.forumriskmanagement.it/images/FORUMRISK11/LABORATORIO-PDTA/REGIONI/LAZIO/bambingesu-RB.pdf.

[17] Houston S.K., Murray T.G., Wolfe S.Q., Fernandes C.E. (2011). Current update on retinoblastoma. Int. Ophthalmol. Clin..

[18] Cunha-Vaz J., Bernardes R., Lobo C. (2011). Blood-retinal barrier. Eur. J. Ophthalmol..

[19] Balderrama J., Carlos Leal-Leal A., Alvis-Miranda H., Lee A. (2013). Intraarterial chemotherapy for retinoblastoma: A practical review. Rom. Neurosurg..

[20] Go-bin Y.P., Dunkel I.J., Marr B.P., Brodie S.E., Abramson D.H. (2011). Intra-arterial chemotherapy for the management of retinoblastoma: Four-year experience. Arch. Ophthalmol..

[21] Suzuki S., Yamane T., Mohri M., Kaneko A. (2011). Selective ophthalmic arterial injection ther-apy for intraocular retinoblastoma: The long-term prognosis. Ophthalmology.

[22] Dimaras H., Corson T.W., Cobrinik D., White A., Zhao J., Munier F.L., Abramson D.H., Shields C.L., Chantada G.L., Njuguna F. (2015). Retinoblastoma. Nat. Rev. Dis. Primers.

[23] Abramson D.H., Dunkel I.J., Brodie S.E., Kim J.W., Gobin Y.P. (2008). A phase I/II study of direct in-traarterial (ophthalmic artery) chemotherapy with melphalan for intraocular retinoblastoma initial results. Ophthalmology.

[24] Shields C.L., Manjandavida F.P., Lally S.E., Pieretti G., Arepalli S.A., Caywood E.H., Jabbour P., Shields J.A. (2014). Intra-arterial chemotherapy for retinoblastoma in 70 eyes: Outcomes based on the international classification of retinoblastoma. Ophthalmology.

[25] Shields C.L., Shields J.A. (2010). Retinoblastoma management: Advances in enucleation, intravenous chemoreduction, and intra-arterial chemotherapy. Curr. Opin. Ophthalmol..

[26] Abramson D.H., Ellsworth R.M., Tretter P., Adams K., Kitchin F.D. (1981). Simultaneous Bilateral Radiation for Advanced Bilateral Retinoblastoma. Arch. Ophthalmol..

[27] Warda O., Naeem Z., Roelofs K.A., Sagoo M.S., Reddy M.A. (2022). Retinoblastoma and vision. Eye.

[28] Gombos D.S., Hungerford J., Abramson D.H., Kingston J., Chantada G., Dunkel I.J., Antoneli C.B.G., Greenwald M., Haik B.G., Leal C.A. (2007). Secondary Acute Myelogenous Leukemia in Patients with Retinoblastoma. Is Chemotherapy a Factor?. Ophthalmology.

[29] Wong F.L., Boice J.D., Abramson D.H., Tarone R.E., Kleinerman R.A., Stovall M., Goldman M.B., Seddon J.M., Tarbell N., Fraumeni J.F. (1997). Cancer incidence after retinoblastoma: Radiation dose and sarcoma risk. JAMA.

[30] Qaddoumi I., Bass J., Wu J., Billups C.A., Wozniak A.W., Merchant T.E., Haik B.G., Wilson M.W., Rodriguez-Galindo C. (2012). Carboplatin-Associated Ototoxicity in Children with Retinoblastoma. J. Clin. Oncol..

[31] Francis J.H., Levin A.M., Zabor E.C., Gobin Y.P., Abramson D.H. (2018). Ten-year experience with ophthalmic artery chemosurgery: Ocular and recurrence-free survival. PLoS ONE.

[32] Abramson D.H., Ji X., Francis J.H., Catalanotti F., Brodie S.E., Habib L. (2019). Intravitreal chemotherapy in retinoblastoma: Expanded use beyond intravitreal seeds. Br J. Ophthalmol..

[33] Schaiquevich P., Francis J.H., Cancela M.B., Montero Carcaboso A., Chantada G.L., Abramson D.H. (2022). Treatment of Retinoblastoma: What Is the Latest and What Is the Future. Front. Oncol..

[34] https://www.cancer.gov/types/retinoblastoma/hp/retinoblastoma-treatment-pdq.

[35] Cancela M.B., Zugbi S., Winter U., Martinez A.L., Sampor C., Sgroi M., Francis J.H., Garippa R., Abramson D.H., Chantada G. (2020). A Decision Process for Drug Discovery in Retinoblastoma. Investig. New Drugs.

[36] Pascual-Pasto G., Olaciregui N.G., Vila-Ubach M., Paco S., Monterrubio C., Rodriguez E., Winter U., Batalla-Vilacis M., Catala J., Salvador H. (2016). Preclinical Platform of Retinoblastoma Xenografts Recapitulating Human Disease and Molecular Markers of Dissemination. Cancer Lett..

[37] Togashi K., Okada M., Suzuki S., Sanomachi T., Seino S., Yamamoto M., Yamashita H., Kitanaka C. (2020). Inhibition of retinoblastoma cell growth by CEP1347 through activation of the P53 pathway. Anticancer. Res..

[38] Gomatou G., Trontzas I., Ioannou S., Drizou M., Syrigos N., Kotteas E. (2021). Mechanisms of resistance to cyclin-dependent kinase 4/6 inhibitors. Mol. Biol. Rep..

[39] vvv.clinicaltrials.gov.

[40] Combs S.S., Lee S.S., Jame A. (2017). Mechanisms of therapeutic CDK4/6 inhibition in breast cancer. Semin. Oncol..

[41] Sherr C.J., Beach D., Shapiro G.I. (2016). Targeting CDK4 and CDK6: From Discovery to Therapy. Cancer Discov..

[42] Hamilton E., Infante J.R. (2016). Targeting CDK4/6 in patients with cancer. Cancer Treat. Rev..

[43] Harada T., Ijima A. (2018). Pharmacological profile and clinical findings of palbociclib. Nippon. Yakurigaku Zasshi.

[44] Pardee A.B., Li Y.Z., Li C.J. (2002). Cancer therapy with beta-lapachone. Curr. Cancer Drug Targets..

[45] Shah M., Murad W., Mubin S., Ullah O., Rehman N.U., Rahman H.M. (2022). Multiple health benefits of curcumin and its therapeutic potential. Environ. Sci. Pollut. Res..

[46] Moballegh N.M., Varzandeh M., Pahlavanneshan S., Mohamadi N., Sarhadi S., Samareh Fekri H., Mohammadinejad R., Ahn K.S. (2022). Curcumin: A potential therapeutic natural product for adenocarcinomas. Phytochem. Lett..

[47] Nair A., Thevenot P., Hu W., Tang L. (2008). Nanotechnology in the treatment and detection of intraocular cancers. J. Biomed. Nanotechnol..

[48] Bhavsar D., Subramanian K., Sethuraman S., Krishnan U.M. (2016). Management of retinoblastoma: Opportunities and challenges. Drug Deliv..

[49] Diebold Y., Calonge M. (2010). Applications of nanoparticles in ophthalmology. Prog. Retin. Eye Res..

[50] Vanderwoot J., Ludwig A. (2007). Ocular drug delivery: Nanomedicines application. Nanomedicine.

[51] Sahoo S.K., Parveen S., Panda J.J. (2007). The present and future of nanotechnology in human health care. Nanomedicine.

[52] Yang C., Lin Z.-I., Chen J.-A., Xu Z., Gu J., Law W.-C., Yang J.H.C., Chen C.-K. (2022). Organic/inorganic self-assembled hybridnano-architectures for cancer therapy applications. Macromol. Biosci..

[53] Peng T., Xu T., Liu X. (2022). Research progress of the engagement of inorganic nanomaterials in cancer immunotherapy. Drug Deliv..

[54] Russo E., Spallarossa A., Tasso B., Villa C., Brullo C. (2021). Nanotechnology of tyrosine kinase inhibitors in cancer therapy: A perspective. Int. J. Mol. Sci..

[55] Moradi S., Mokhtari-Dizaji M., Ghassemi F., Sheibani S., Amoli F.A. (2020). The effect of ultrasound hyperthermia with gold nanoparticles on retinoblastoma Y79 cells. Gold Bull..

[56] Wang M., Yang Q., Li M., Zou H., Wang Z., Ran H., Zheng Y., Jian J., Zhou Y., Luo Y. (2020). Multifunctional nanoparticles for multimodal imaging-guided low-intensity focused ultrasound/immunosynergistic retinoblastoma therapy. ACS Appl. Mater. Interfaces.

[57] Darviot C., Hardy P., Meunier M. (2019). Laser-induced plasmon-mediated treatment of retinoblastoma in viscous vitreous phantom. J. Biophotonics.

[58] Kalmodia S., Parameswaran S., Ganapathy K., Yang W., Barrow C.J., Kanwar J.R., Roy K., Vasudevan M., Kulkarni K., Elchuri S.V. (2017). Characterization and molecular mechanism of peptide-conjugated gold nanoparticle inhibiting p53-hdm2 interaction in retinoblastoma. Mol. Ther. Nucleic Acids.

[59] Mitra M., Kandalam M., Rangasamy J., Shankar B., Maheswari U.K., Swaminathan S., Krishnakumar S. (2013). Novel epithelial cell adhesion molecule antibody conjugated polyethyleneimine-capped gold nanoparticles for enhanced and targeted small interfering RNA delivery to retinoblastoma cells. Mol. Vis..

[60] Rajanahalli P., Stucke C.J., Hong Y. (2015). The effects of silver nanoparticles on mouse embryonic stem cell self-renewal and proliferation. Toxicol. Rep..

[61] Demirci H., Slimani N., Pawar M., Kumon R.E., Vaishnava P., Besirli C.G. (2019). Magnetic hyperthermia in y79 retinoblastoma and ARPE-19 retinal epithelial cells: Tumor selective apoptotic activity of iron oxide nanoparticle. Trans. Vis. Sci. Technol..

[62] Sapareto S.A., Dewey W.C. (1984). Thermal dose determination in cancer therapy. Int. J. Radiat Oncol. Biol. Phys..

[63] Qu W., Meng B., Yu Y., Wang S. (2017). EpCAM antibody-conjugated mesoporous silica nanoparticles to enhance the anticancer efficacy of carboplatin in retinoblastoma. Mater. Sci. Eng. C.

[64] Gallud A., Warther D., Maynadier M., Sefta M., Poyer F., Thomas C.D., Rouxel C., Mongin O., Blanchard-Desce M., Morère A. (2015). Identification of MRC2 and CD209 receptors as targets for photodynamic therapy of retinoblastoma using mesoporous silica nanoparticles. RSC Adv..

[65] Gary-Bobo M., Mir Y., Rouxel C., Brevet D., Hocine O., Maynadier M., Gallud A., Da Silva A., Mongin O., Blanchard-Desce M. (2012). Multifunctionalized mesoporous silica nanoparticles for the in vitro treatment of retinoblastoma: Drug delivery, one and two-photon photodynamic therapy. Int. J. Pharm..

[66] Warther D., Mauriello Jimenez C., Raehm L., Gerardin C., Durand J.-O., Morère A., El Cheikh K., Gallud A., Gary-Bobo M., Maynadier M. (2014). Small sized mesoporous silica nanoparticles functionalized with mannose for retinoblastoma cell imaging. RSC Adv..

[67] Deshpande S., Patil S., Kuchibhatla S.V.N.T., Seal S. (2005). Size dependency variation in lattice parameter and valency states in nanocrystalline cerium oxide. Appl. Phys. Lett..

[68] Chen B.-H., Inbaraj B.S. (2018). Various physicochemical and surface properties controlling the bioactivity of cerium oxide nanoparticles. Crit. Rev. Biotechnol..

[69] Inbaraj B.S., Chen B.-H. (2020). An overview on recent in vivo biological application of cerium oxide nanoparticles. Asian J. Pharm..

[70] Gao R., Mitra R.N., Zheng M., Wang K., Dahringer J.C., Han Z. (2018). Developing nanoceria-based pH-dependent cancer-directed drug delivery system for retinoblastoma. Adv. Funct. Mater..

[71] Kartha B., Thanikachalam K., Vijayakumar N., Alharbi N.S., Kadaikunnan S., Khaled J.M., Gopinath K., Govindarajan M. (2022). Synthesis and characterization of Ce-doped TiO2 nanoparticles and their enhanced anticancer activity in Y79 retinoblastoma cancer cells. Green Process. Synth..

[72] Mitra R.N., Gao R., Zheng M., Wu M.J., Voinov M.A., Smirnov A.I., Smirnova T.I., Wang K., Chavala S., Han Z. (2017). Glycol chitosan engineered autoregenerative antioxidant significantly attenuates pathological damages in models of age-related macular degeneration. ACS Nano.

[73] Rodríguez-Nogales C., Gonzalez-Fernandez Y., Aldaz A., Couvreur P., Blanco-Prieto M.J. (2018). Nanomedicines for Pediatric Cancers. ACS Nano.

[74] Liu S., Jones L., Gu F.X. (2012). Nanomaterials for ocular drug delivery. Macromol. Biosci..

[75] Chaurasia S.S., Lim R.R., Lakshminarayanan R., Mohan R.R. (2015). Nanomedicine approaches for corneal diseases. J. Funct. Biomater..

[76] Mudigunda S.V., Pemmaraju D.B., Paradkar S., Puppala E.R., Gawali B., Upadhyayula S.M., Gangamodi N.V., Rengan A.K. (2022). Multifunctional polymeric nanoparticles for chemo/phototheranostics of retinoblastoma. ACS Biomater. Sci. Eng..

[77] Godse R., Rathod M., De A., Shinde U. (2021). Intravitreal galactose conjugated polymeric nanoparticles of etoposide for retinoblastoma. J. Drug Deliv. Sci. Technol..

[78] Sims L.B., Tyo K.M., Stocke S., Mahmoud M.Y., Ramasubramanian A., Steinbach-Rankins J.M. (2019). Surface-modified melphalan nanoparticles for intravitreal chemotherapy of retinoblastoma. IOVS.

[79] Inomata M., Kaneko A. (1987). Chemosensitivity profiles of primary and cultured human retinoblastoma cells in a human tumor clonogenic assay. Jpn. J. Cancer Res..

[80] Silva-Abreu M., Calpena A.C., Espina M., Silva A.M., Gimeno A., Egea M.A., García M.L. (2018). Optimization, biopharmaceutical profile and therapeutic efficacy of pioglitazone-loaded PLGA-PEG nanospheres as a novel strategy for ocular inflammatory disorders. Pharm. Res..

[81] Delrish E., Ghassemi F., Jabbarvand M., Lashay A., Atyabi F., Soleimani M., Dinarvand R. (2022). Biodistribution of cy5-labeled thiolated and methylated chitosan-carboxymethyl dextran nanoparticles in an animal model of retinoblastoma. J. Ophthalmic Vis. Res..

[82] Delrish E., Jabbarvand M., Ghassemi F., Amoli F.A., Atyabi F., Lashay A., Soleimani M., Aghajanpour L., Dinarvand R. (2021). Efficacy of topotecan nanoparticles for intravitreal chemotherapy of retinoblastoma. Exp. Eye Res..

[83] Passos Gibson V., Derbali R.M., Phan H.T., Tahiri H., Allen C., Hardy P., Chain J.L. (2020). Survivin silencing improved the cytotoxicity of carboplatin and melphalan in Y79 and primary retinoblastoma cells. Int. J. Pharm..

[84] Tabatabaei S.N., Derbali R.M., Yang C., Superstein R., Hamel P., Chain J.L., Hardy P. (2019). Co-delivery of miR-181a and melphalan by lipid nanoparticles for treatment of seeded retinoblastoma. J. Control. Release.

[85] Narayana R.V.L., Jana P., Tomar N., Prabhu V., Nair R.M., Manukonda R., Kaliki S., Coupland S.E., Alexander J., Kalirai H. (2021). Carboplatin and etoposide loaded lactoferrin protein nanoparticles for targeting cancer stem cells in retinoblastoma in vitro. IOVS.

[86] Ahmeda F., Ali M.J., Kondapi A.K. (2014). Carboplatin loaded protein nanoparticles exhibit improve anti-proliferative activity in retinoblastoma cells. Int. J. Biol. Macromol..

[87] Shome D., Kalita D., Jain V., Sarin R., Maru G.B., Bellare J.R. (2014). Carboplatin loaded polymethylmethacrylate nanoparticles in an adjunctive role in retinoblastoma: An animal trial. Indian J. Ophtalm..

[88] Kalita D., Shome D., Jain V.G., Chadha K., Bellare J.R. (2014). In vivo intraocular distribution and safety of periocular nanoparticle carboplatin for treatment of advanced retinoblastoma in humans. Am. J. Ophthalmol..

[89] Alsaab H., Alzhrani R.M., Kesharwani P., Sau S., Boddu S.H.S., Iyer A.K. (2017). Folate decorated nanomicelles loaded with a potent curcumin analogue for targeting retinoblastoma. Pharmaceutics.

[90] Li Z., Wu X., Li Lin Yao J., Sun L., Shi Y., Zhang W., Lin J., Liang D., Li Y. (2012). Antitumor activity of celastrol nanoparticles in a xenograft retinoblastoma tumor model. Int. J. Nanomed..

[91] Guo Z., Shi L., Feng H., Yang F., Li Z., Zhang J., Jin L., Li J. (2021). Reduction-sensitive nanomicelles: Delivery celastrol for retinoblastoma cells effective apoptosis. Chin. Chem. Lett..

[92] Li Z., Guo Z., Chu D., Feng H., Zhang J., Zhu L., Li J. (2020). Effectively suppressed angiogenesis-mediated retinoblastoma growth using celastrol nanomicelles. Drug Deliv..

[93] Silva A.M., Alvarado H.L., Abrego G., Martins-Gomes C., Garduño-Ramirez M.L., García M.L., Calpena A.C., Souto E.B. (2019). In vitro cytotoxicity of oleanolic/ursolic acids loaded in PLGA nanoparticles in different cell lines. Pharmaceutics.

[94] N’Diaye M., Vergnaud-Gauduchon J., Nicolas V., Faure V., Denis S., Abreu S., Chaminade P., Rosilio V. (2019). Hybrid lipid polymer nanoparticles for combined chemo- and photodynamic therapy. Mol. Pharm..

[95] Remya R.R., Radhika Rajasree S.R., Suman T.Y., Aranganathan L., Gayathri S., Gobalakrishnan M., Karthih M.G. (2018). Laminarin based AgNPs using brown seaweed *Turbinaria ornata* and its induction of apoptosis in human retinoblastoma Y79 cancer cell lines. Mater. Res. Express.

[96] Remya R.R., Radhika Rajasree S.R., Aranganathan L., Suman T.Y., Gayathri S. (2017). Enhanced cytotoxic activity of AgNPs on retinoblastoma Y79 cell lines synthesised using marine seaweed *Turbinaria ornata*. IET Nanobiotechnol..

[97] Zou H., Li M., Li X., Zheng W., Kuang H., Wang M., Zhang W., Ran H., Ma H., Zhou X. (2022). Multimodal imaging and photothermal synergistic immunotherapy of retinoblastoma with tuftsin-loaded carbonized MOF nanoparticles. Drug Deliv..

[98] Li M., Bian X., Chen X., Fan N., Zou H., Bao Y., Zhou Y. (2022). Multifunctional liposome for photoacoustic/ultrasound imaging-guided chemo/photothermal retinoblastoma therapy. Drug Deliv..

[99] Tang C., Li L., Mo T., Na J., Qian Z., Fan D., Sun X., Yao M., Pan L., Huang Y. (2022). Oncolytic viral vectors in the era of diversifed cancer therapy: From preclinical to clinical. Clin. Transl. Oncol..

[100] Pierce K.M., Miklavcic W.R., Cook K.P., Hennen M.S., Bayles K.W., Hollingsworth M.A., Brooks A.E., Pullan J.E., Dailey K.M. (2021). The Evolution and Future of Targeted Cancer Therapy: From Nanoparticles, Oncolytic Viruses, and Oncolytic Bacteria to the Treatment of Solid Tumors. Nanomaterials.

[101] Dick F.A., Rubin S.M. (2013). Molecular mechanisms underlying RB protein function. Nat. Rev. Mol. Cell Biol..

[102] Pascual-Pasto G., Bazan-Peregrino M., Olaciregui N.G., Restrepo-Perdomo C.A., Mato-Berciano A., Ottaviani D., Weber K., Correa G., Paco S., Vila-Ubach M. (2019). Therapeutic targeting of the RB1 pathway in retinoblastoma with the oncolytic adenovirus VCN-01. Sci. Transl. Med..

[103] Koch J., Schober S.J., Hindupur S.V., Schöning C., Klein F.G., Mantwill K., Ehrenfeld M., Schillinger U., Hohnecker T., Qi P. (2022). Targeting the Retinoblastoma/E2F repressive complex by CDK4/6 inhibitors amplifies oncolytic potency of an oncolytic adenovirus. Nat. Commun..

[104] Suryawanshi Y.R., Zhang T., Razi F., Essani K. (2020). Tanapoxvirus: From discovery towards oncolytic immunovirotherapy. J. Cancer Res. Ther..

[105] VanDeusen H.R., Kalejta R.F. (2015). The Retinoblastoma Tumor Suppressor Promotes Efficient Human Cytomegalovirus Lytic Replication. J. Virol..

